# Crystal structure of *cis*,*fac*-{*N*,*N*-bis­[(pyridin-2-yl)meth­yl]methyl­amine-κ^3^ *N*,*N*′,*N*′′}di­chlorido­(dimethyl sulfoxide-κ*S*)ruthenium(II)

**DOI:** 10.1107/S2056989015014875

**Published:** 2015-08-22

**Authors:** Kasey Trotter, Navamoney Arulsamy, Elliott Hulley

**Affiliations:** a University of Wyoming, 1000 E University Ave, Dept. 3838, Laramie, WY 82071, USA

**Keywords:** crystal structure, ruthenium(II) complex, S-bound dimethyl sulfoxide, distorted octa­hedral coordination geometry

## Abstract

The reaction of di­chlorido­tetra­kis­(dimethyl sulfoxide)­ruthen­ium(II) with *N*,*N*-bis[(pyridin-2-yl)meth­yl]methyl­amine aff­ords the title complex, [RuCl_2_(C_13_H_15_N_3_)(C_2_H_6_OS)]. The asymmetric unit contains a well-ordered complex mol­ecule. The *N*,*N*-bis­[(pyridin-2-yl)meth­yl]methyl­amine (bpma) ligand binds the cation through its two pyridyl N atoms and one aliphatic N atom in a facial manner. The coordination sphere of the low-spin *d* ^6^ Ru^II^ is distorted octahedral. The dimethyl sulfoxide (dmso) ligand coordinates to the cation through its S atom and is *cis* to the aliphatic N atom. The two chloride ligands occupy the remaining sites. The bpma ligand is folded with the dihedral angle between the mean planes passing through its two pyridine rings being 64.55 (8)°. The two N—Ru—N bite angles of the ligand at 81.70 (7) and 82.34 (8)° illustrate the distorted octa­hedral coordination geometry of the Ru^II^ cation. Two neighboring molecules are weakly associated through mutual intermolecular hydrogen bonding involving the O atom and one of the methyl groups of the dmso ligand. One of the chloride ligands is also weakly hydrogen bonded to a pyridyl H atom of another molecule.

## Related literature   

For the synthesis of bpma, see: Astner *et al.* (2008 ▸). For the synthesis of RuCl_2_(dmso)_4_ (dmso is dimethyl sulfoxide), see: Evans *et al.* (1973 ▸). Ruthenium(II) complexes of pyridine-based ligands which also contain a dmso ligand act as catalytic initiators (Bressan & Morvillo, 1992 ▸; Carvalho *et al.*, 2014 ▸; Ferrer *et al.*, 2013 ▸). The ambidentate dmso ligand exhibits preferential binding through its S atom with low-spin *d* ^6^ Ru^II^ cations and through its O atom with Ru^III^ cations (Roeser *et al.*, 2013 ▸; Smith *et al.*, 2000 ▸). Ruthenium(II) complexes containing the labile dmso and chloride ligands are particularly attractive precursors for the synthesis of specifically designed catalysts. For the synthesis and structures of such complexes, see: Fischer *et al.* (2009 ▸); Mola *et al.* (2007 ▸). For complexes containing facially coordinated tridentate ligands, see: Dakkach *et al.* (2013 ▸); Fischer *et al.* (2009 ▸); Mishra *et al.* (2009 ▸); Matsuya *et al.* (2009 ▸); Mola *et al.* (2006 ▸, 2007 ▸, 2009 ▸); Rodriguez *et al.* (2001 ▸); Sala *et al.* (2008 ▸); Serrano *et al.* (2006 ▸); Shimizu *et al.* (2008 ▸); Suzuki *et al.* (2014 ▸)
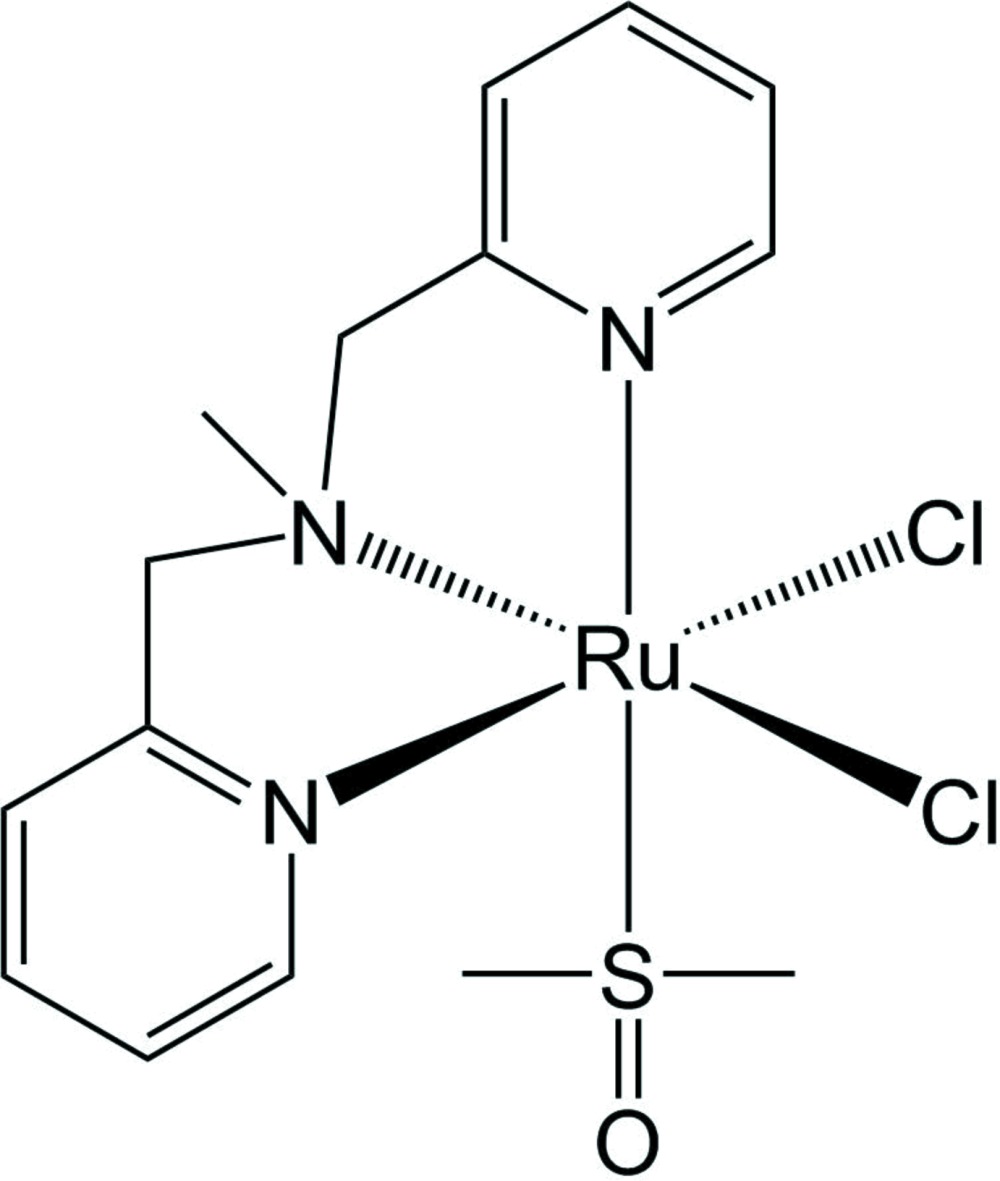


## Experimental   

### Crystal data  

[RuCl_2_(C_13_H_15_N_3_)(C_2_H_6_OS)]
*M* *_r_* = 463.38
Monoclinic, 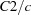

*a* = 14.6117 (3) Å
*b* = 9.3345 (2) Å
*c* = 27.3451 (7) Å
β = 102.734 (1)°
*V* = 3637.94 (14) Å^3^
*Z* = 8
Mo *K*α radiation
μ = 1.28 mm^−1^
*T* = 150 K
0.21 × 0.17 × 0.11 mm

### Data collection  

Bruker APEXII CCD diffractometer
Absorption correction: multi-scan (*SAINT*; Bruker, 2009 ▸) *T*_min_ = 0.647, *T*_max_ = 0.747
33234 measured reflections
7273 independent reflections
5265 reflections with *I* > 2σ(*I*)
*R*_int_ = 0.068

### Refinement  

*R*[*F* ^2^ > 2σ(*F* ^2^)] = 0.039
*wR*(*F* ^2^) = 0.085
*S* = 1.01
7273 reflections
292 parameters
All H-atom parameters refined
Δρ_max_ = 1.13 e Å^−3^
Δρ_min_ = −0.92 e Å^−3^

### 

Data collection: *APEX2* (Bruker, 2009 ▸); cell refinement: *SAINT* (Bruker, 2009 ▸); data reduction: *SAINT*; program(s) used to solve structure: *SHELXS97* (Sheldrick, 2008 ▸); program(s) used to refine structure: *SHELXL2014* (Sheldrick, 2015 ▸); molecular graphics: *SHELXTL* (Sheldrick, 2008 ▸); software used to prepare material for publication: *SHELXTL*.

## Supplementary Material

Crystal structure: contains datablock(s) New_Global_Publ_Block, I. DOI: 10.1107/S2056989015014875/zq2232sup1.cif

Structure factors: contains datablock(s) I. DOI: 10.1107/S2056989015014875/zq2232Isup2.hkl

Structural commentary. DOI: 10.1107/S2056989015014875/zq2232sup3.docx

Supporting information file. DOI: 10.1107/S2056989015014875/zq2232sup3.pdf

**Fig. 1.**
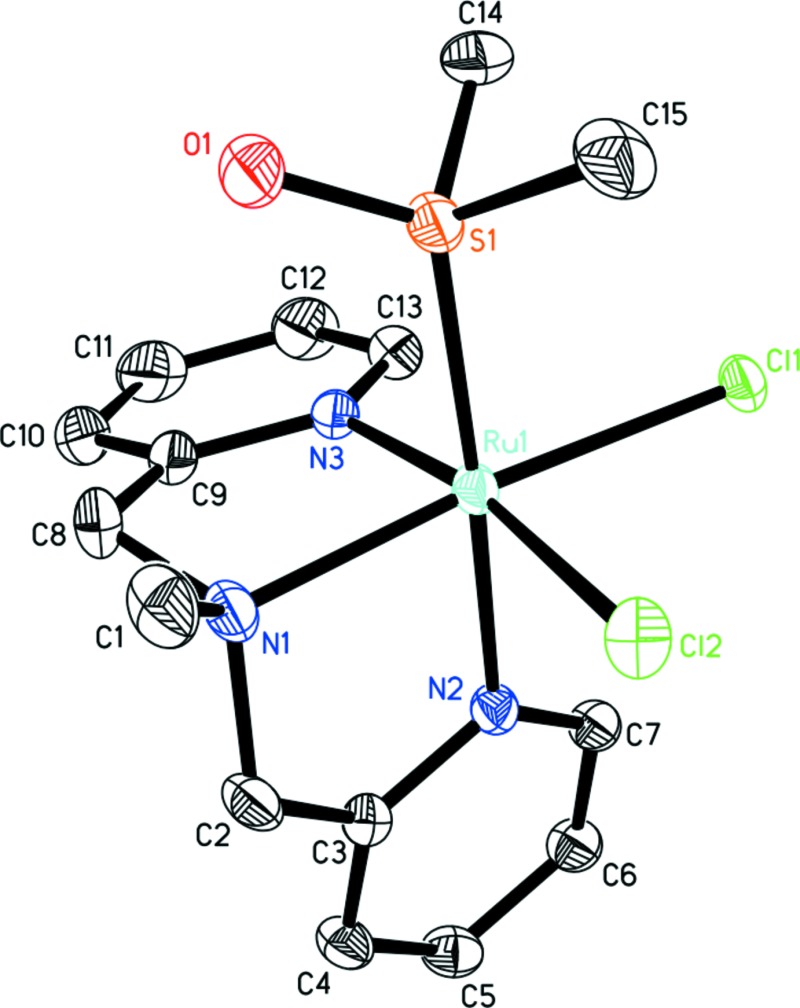
View of RuCl2(dpma)(dmso). H atoms have been omitted and displacement parameters are drawn at the 50% probability level.

2 . DOI: 10.1107/S2056989015014875/zq2232fig1.tif
View of RuCl_2_(dpma)(dmso). H atoms have been omitted and displacement parameters are drawn at the 50% probability level.

CCDC reference: 1417672

Additional supporting information:  crystallographic information; 3D view; checkCIF report

## Figures and Tables

**Table 1:** Hydrogen-bond geometry (, )

*D*H*A*	*D*H	H*A*	*D* *A*	*D*H*A*
C14H14*A*O1^i^	0.91(4)	2.54(4)	3.431(3)	169(3)
C4H4Cl1^ii^	0.93(3)	2.58(3)	3.487(2)	165(2)
C1H1*C*O1	0.99(3)	2.32(3)	3.182(4)	145(2)

Symmetry codes: (i) 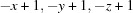; (ii) 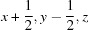.

## supplementary crystallographic information

### S1. Comment

Ruthenium(II) complexes of pyridine-based ligands which also contain a di­methyl­sulfoxide (dmso) ligand act as catalytic initiators (Bressan & Morvillo, 1992; Carvalho *et al.*, 2014; Ferrer *et al.*, 2013). The ambidentate dmso appears to show preferential binding through its S atom with Ru^II^ centers, and its O atom with Ru^III^ centers (Roeser *et al.*, 2013; Smith *et al.*, 2000). Ruthenium(II) complexes containing the labile dmso and chloride ligands are particularly attractive precursors for the synthesis of specifically-designed catalysts. Our research project is aimed at the catalytic reduction of stable anions such as perchlorates using Ru^II^ precatalysts. Multidentate ligands are expected to stabilize ruthenium(IV)–oxido inter­mediates suggested as inter­mediates in the catalytic oxidation of a variety of organic substrates in the presence of hypochlorite, perchlorate and other oxidizers (Bressan & Morvillo 1992). Here we report the X-ray crystal structural determination of a potential precursor ruthenium complex. The title compound, RuCl_2_(bpma)(dmso), is synthesized from the reaction of RuCl_2_(dmso)_4_ (Evans *et al.*, 1973 ) with *N*,*N*-bis­(pyridin-2-yl­methyl)­methyl­amine (bpma) (Astner *et al.*, 2008).

### S2. Structural commentary

The asymmetric unit contains a well-ordered RuCl_2_(bpma)(dmso) molecule. The metal center is in a distorted o­cta­hedral geometry with the tridentate bpma ligand binding through its two pyridyl N atoms and aliphatic N atom in a facial mode, as shown in Fig. 1. The two chloride ligands occupy two adjacent sites, and the dmso ligand is present trans to one of the pyridyl N atoms. The tridentate ligand is folded to achieve facial coordination, and the extent of folding is reflected in the small dihedral angle of 64.55 (8)° between the mean planes passing through the two pyridine rings. The two N—Ru—N bite angles of the ligand at 81.70 (7) and 82.34 (8)° are illustrative of the distorted o­cta­hedral geometry of the metal center. The complex can be represented as the *cis*,*fac*-isomer to indicate the *cis*-geometry of the dmso ligand to the aliphatic N atom and the facial coordination mode of bpma. A literature survey of Ru^II^ complexes of bpma and those of closely related bis­(pyridin-2-yl­methyl)­alkyl­amine ligands reveals that an overwhelming majority of the complexes contain facially coordinated tridentate ligands (Dakkach *et al.*, 2013; Fischer *et al.*, 2009; Mishra *et al.*, 2009; Matsuya *et al.*, 2009; Mola *et al.*, 2006, 2007, 2009; Rodriguez *et al.*, 2001; Sala *et al.*, 2008; Serrano *et al.*, 2006; Shimizu *et al.*, 2008; Suzuki *et al.*, 2014). *cis*,*fac*-isomer is the thermodynamically favored (Mola *et al.*, 2007), and therefore the more frequent occurrence of this isomer is unsurprising. However, Shimuzu *et al.* suggest that the binding mode of the tridentate ligand depends on the nature of the other ligands with the hydroxo and methoxo ligands favoring meridional coordination mode for the tridentate ligands (Shimizu *et al.*, 2008). The Ru—N_py_ distances in the present complex are unequal as they have either a chloride or dmso ligands in their respective *trans* positions. The Ru—dmso bond is unexceptional at 2.2207 (6) Å, and comparable to those found in *cis*,*fac*-RuCl_2_(bpma)(dmso) and *trans*,*mer*-RuCl_2_(bpea)(dmso) (Mola *et al.*, 2007).

### Crystal data

**Table:**

[RuCl_2_(C_13_H_15_N_3_)(C_2_H_6_OS)]	*F*(000) = 1872
*M**_r_* = 463.38	*D*_x_ = 1.692 Mg m^−^^3^
Monoclinic, *C*2/*c*	Mo *K*α radiation, λ = 0.71073 Å
*a* = 14.6117 (3) Å	Cell parameters from 4846 reflections
*b* = 9.3345 (2) Å	θ = 2.6–29.0°
*c* = 27.3451 (7) Å	µ = 1.28 mm^−^^1^
β = 102.734 (1)°	*T* = 150 K
*V* = 3637.94 (14) Å^3^	Rectangular, yellow
*Z* = 8	0.21 × 0.17 × 0.11 mm

### Data collection

**Table:**

Bruker APEXII CCD diffractometer	5265 reflections with *I* > 2σ(*I*)
φ and ω scans	*R*_int_ = 0.068
Absorption correction: multi-scan (*SAINT*; Bruker, 2009)	θ_max_ = 33.7°, θ_min_ = 2.6°
*T*_min_ = 0.647, *T*_max_ = 0.747	*h* = −19→22
33234 measured reflections	*k* = −14→14
7273 independent reflections	*l* = −42→42

### Refinement

**Table:**

Refinement on *F*^2^	0 restraints
Least-squares matrix: full	Hydrogen site location: difference Fourier map
*R*[*F*^2^ > 2σ(*F*^2^)] = 0.039	All H-atom parameters refined
*wR*(*F*^2^) = 0.085	*w* = 1/[σ^2^(*F*_o_^2^) + (0.0338*P*)^2^] where *P* = (*F*_o_^2^ + 2*F*_c_^2^)/3
*S* = 1.01	(Δ/σ)_max_ = 0.002
7273 reflections	Δρ_max_ = 1.13 e Å^−^^3^
292 parameters	Δρ_min_ = −0.92 e Å^−^^3^

### Special details

**Table:**

Geometry. All e.s.d.'s (except the e.s.d. in the dihedral angle between two l.s. planes) are estimated using the full covariance matrix. The cell e.s.d.'s are taken into account individually in the estimation of e.s.d.'s in distances, angles and torsion angles; correlations between e.s.d.'s in cell parameters are only used when they are defined by crystal symmetry. An approximate (isotropic) treatment of cell e.s.d.'s is used for estimating e.s.d.'s involving l.s. planes.

### Fractional atomic coordinates and isotropic or equivalent isotropic displacement parameters (Å^2^)

**Table:**

	*x*	*y*	*z*	*U*_iso_*/*U*_eq_	
Ru1	0.67345 (2)	0.51144 (2)	0.37644 (2)	0.01639 (5)	
Cl1	0.54447 (4)	0.65278 (5)	0.33000 (2)	0.02026 (11)	
Cl2	0.77049 (4)	0.72523 (6)	0.39465 (2)	0.02641 (13)	
N1	0.78992 (14)	0.3807 (2)	0.40882 (7)	0.0224 (4)	
N2	0.72321 (14)	0.47141 (18)	0.31136 (7)	0.0169 (3)	
N3	0.61032 (14)	0.31637 (19)	0.35845 (7)	0.0195 (4)	
S1	0.61157 (5)	0.53018 (6)	0.44340 (2)	0.02421 (13)	
O1	0.64939 (15)	0.4440 (2)	0.48897 (7)	0.0394 (5)	
C14	0.4890 (2)	0.4893 (3)	0.42722 (10)	0.0321 (6)	
H14A	0.459 (3)	0.515 (3)	0.4517 (13)	0.045 (10)*	
H14B	0.461 (2)	0.541 (3)	0.3971 (12)	0.038 (8)*	
H14C	0.485 (2)	0.388 (4)	0.4213 (11)	0.042 (9)*	
C15	0.6052 (2)	0.7116 (3)	0.46376 (11)	0.0346 (6)	
H15A	0.571 (2)	0.713 (3)	0.4902 (11)	0.037 (8)*	
H15B	0.673 (3)	0.748 (4)	0.4742 (12)	0.063 (11)*	
H15C	0.576 (2)	0.775 (3)	0.4363 (10)	0.033 (8)*	
C1	0.8474 (2)	0.4251 (3)	0.45892 (9)	0.0308 (6)	
H1A	0.8987 (18)	0.356 (3)	0.4712 (9)	0.018 (6)*	
H1B	0.873 (2)	0.523 (3)	0.4570 (11)	0.033 (8)*	
H1C	0.805 (2)	0.427 (3)	0.4826 (10)	0.028 (7)*	
C2	0.85411 (17)	0.3835 (3)	0.37313 (8)	0.0235 (5)	
H2A	0.8994 (19)	0.303 (3)	0.3795 (9)	0.027 (7)*	
H2B	0.891 (2)	0.477 (3)	0.3815 (11)	0.030 (8)*	
C3	0.80268 (16)	0.3934 (2)	0.31945 (8)	0.0181 (4)	
C4	0.83608 (18)	0.3340 (2)	0.28026 (9)	0.0221 (5)	
H4	0.892 (2)	0.283 (3)	0.2870 (9)	0.025 (7)*	
C5	0.78772 (18)	0.3565 (2)	0.23159 (9)	0.0230 (5)	
H5	0.8051 (18)	0.312 (3)	0.2059 (9)	0.022 (7)*	
C6	0.70671 (17)	0.4370 (3)	0.22333 (8)	0.0220 (5)	
H6	0.6718 (19)	0.455 (3)	0.1914 (10)	0.024 (7)*	
C7	0.67585 (17)	0.4919 (2)	0.26393 (8)	0.0189 (4)	
H7	0.617 (2)	0.545 (3)	0.2603 (10)	0.027 (7)*	
C8	0.7522 (2)	0.2349 (3)	0.41426 (10)	0.0293 (6)	
H8A	0.736 (2)	0.230 (3)	0.4470 (11)	0.041 (9)*	
H8B	0.795 (2)	0.167 (3)	0.4131 (10)	0.027 (7)*	
C9	0.66441 (17)	0.2018 (2)	0.37632 (8)	0.0231 (5)	
C10	0.6356 (2)	0.0633 (3)	0.36206 (11)	0.0315 (6)	
H10	0.674 (2)	−0.012 (3)	0.3745 (10)	0.028 (8)*	
C11	0.5508 (2)	0.0411 (3)	0.33028 (12)	0.0355 (7)	
H11	0.532 (2)	−0.043 (4)	0.3154 (12)	0.046 (9)*	
C12	0.4947 (2)	0.1582 (3)	0.31229 (11)	0.0318 (6)	
H12	0.4356 (19)	0.140 (3)	0.2891 (10)	0.024 (7)*	
C13	0.52796 (18)	0.2934 (3)	0.32657 (9)	0.0240 (5)	
H13	0.499 (2)	0.370 (3)	0.3156 (10)	0.029 (7)*	

### Atomic displacement parameters (Å^2^)

**Table:**

	*U*^11^	*U*^22^	*U*^33^	*U*^12^	*U*^13^	*U*^23^
Ru1	0.01879 (10)	0.01715 (8)	0.01358 (7)	0.00499 (7)	0.00432 (6)	0.00194 (6)
Cl1	0.0204 (3)	0.0194 (2)	0.0211 (2)	0.0071 (2)	0.0046 (2)	0.00371 (18)
Cl2	0.0289 (3)	0.0227 (3)	0.0249 (3)	−0.0001 (2)	0.0002 (2)	−0.0007 (2)
N1	0.0228 (11)	0.0253 (9)	0.0193 (8)	0.0090 (8)	0.0051 (7)	0.0030 (7)
N2	0.0187 (9)	0.0165 (8)	0.0160 (8)	0.0026 (7)	0.0049 (7)	0.0007 (6)
N3	0.0237 (10)	0.0179 (8)	0.0193 (8)	0.0050 (7)	0.0099 (7)	0.0034 (7)
S1	0.0300 (3)	0.0280 (3)	0.0163 (2)	0.0123 (2)	0.0088 (2)	0.0041 (2)
O1	0.0504 (13)	0.0511 (12)	0.0208 (8)	0.0271 (10)	0.0167 (8)	0.0156 (8)
C14	0.0352 (16)	0.0393 (15)	0.0273 (12)	0.0084 (12)	0.0185 (11)	0.0049 (11)
C15	0.0433 (18)	0.0339 (14)	0.0287 (13)	0.0102 (13)	0.0123 (13)	−0.0069 (11)
C1	0.0302 (15)	0.0411 (15)	0.0178 (11)	0.0101 (12)	−0.0017 (10)	0.0010 (10)
C2	0.0172 (12)	0.0316 (12)	0.0218 (10)	0.0084 (10)	0.0044 (9)	0.0020 (9)
C3	0.0181 (11)	0.0184 (9)	0.0179 (9)	0.0012 (8)	0.0038 (8)	0.0018 (7)
C4	0.0199 (12)	0.0243 (11)	0.0231 (10)	0.0044 (9)	0.0070 (9)	−0.0015 (8)
C5	0.0240 (13)	0.0254 (11)	0.0217 (10)	0.0001 (9)	0.0093 (9)	−0.0050 (9)
C6	0.0228 (12)	0.0259 (11)	0.0174 (10)	0.0012 (9)	0.0047 (9)	0.0010 (8)
C7	0.0193 (11)	0.0217 (10)	0.0156 (9)	0.0013 (9)	0.0038 (8)	0.0004 (8)
C8	0.0311 (15)	0.0254 (11)	0.0316 (13)	0.0110 (11)	0.0073 (11)	0.0113 (10)
C9	0.0269 (13)	0.0197 (10)	0.0251 (11)	0.0058 (9)	0.0113 (9)	0.0058 (8)
C10	0.0382 (16)	0.0195 (11)	0.0423 (15)	0.0079 (11)	0.0211 (13)	0.0063 (10)
C11	0.0410 (17)	0.0200 (11)	0.0512 (17)	−0.0061 (11)	0.0224 (14)	−0.0047 (11)
C12	0.0298 (15)	0.0251 (12)	0.0414 (15)	−0.0065 (11)	0.0101 (12)	−0.0044 (10)
C13	0.0236 (13)	0.0223 (11)	0.0276 (11)	0.0025 (9)	0.0087 (10)	0.0021 (9)

### Geometric parameters (Å, º)

**Table:**

Ru1—N3	2.0515 (19)	C1—H1C	0.99 (3)
Ru1—N2	2.0989 (18)	C2—C3	1.497 (3)
Ru1—N1	2.1224 (19)	C2—H2A	0.99 (3)
Ru1—S1	2.2207 (6)	C2—H2B	1.02 (3)
Ru1—Cl1	2.4187 (5)	C3—C4	1.387 (3)
Ru1—Cl2	2.4352 (6)	C4—C5	1.378 (3)
N1—C8	1.489 (3)	C4—H4	0.93 (3)
N1—C2	1.495 (3)	C5—C6	1.378 (3)
N1—C1	1.499 (3)	C5—H5	0.90 (2)
N2—C7	1.342 (3)	C6—C7	1.385 (3)
N2—C3	1.347 (3)	C6—H6	0.92 (3)
N3—C13	1.339 (3)	C7—H7	0.97 (3)
N3—C9	1.355 (3)	C8—C9	1.493 (4)
S1—O1	1.4838 (18)	C8—H8A	0.98 (3)
S1—C14	1.788 (3)	C8—H8B	0.89 (3)
S1—C15	1.791 (3)	C9—C10	1.389 (3)
C14—H14A	0.91 (4)	C10—C11	1.364 (4)
C14—H14B	0.96 (3)	C10—H10	0.92 (3)
C14—H14C	0.96 (3)	C11—C12	1.390 (4)
C15—H15A	0.97 (3)	C11—H11	0.90 (3)
C15—H15B	1.03 (4)	C12—C13	1.378 (3)
C15—H15C	0.97 (3)	C12—H12	0.97 (3)
C1—H1A	0.99 (3)	C13—H13	0.85 (3)
C1—H1B	0.99 (3)		
			
N3—Ru1—N2	81.96 (7)	H1A—C1—H1B	110 (2)
N3—Ru1—N1	82.34 (8)	N1—C1—H1C	107.1 (17)
N2—Ru1—N1	81.70 (7)	H1A—C1—H1C	109 (2)
N3—Ru1—S1	91.40 (5)	H1B—C1—H1C	109 (2)
N2—Ru1—S1	173.35 (5)	N1—C2—C3	112.92 (19)
N1—Ru1—S1	97.82 (5)	N1—C2—H2A	111.3 (15)
N3—Ru1—Cl1	95.80 (5)	C3—C2—H2A	113.0 (15)
N2—Ru1—Cl1	91.57 (5)	N1—C2—H2B	104.0 (17)
N1—Ru1—Cl1	173.20 (5)	C3—C2—H2B	107.0 (17)
S1—Ru1—Cl1	88.75 (2)	H2A—C2—H2B	108 (2)
N3—Ru1—Cl2	170.84 (6)	N2—C3—C4	121.8 (2)
N2—Ru1—Cl2	91.40 (5)	N2—C3—C2	114.98 (19)
N1—Ru1—Cl2	90.49 (6)	C4—C3—C2	123.2 (2)
S1—Ru1—Cl2	95.24 (2)	C5—C4—C3	119.5 (2)
Cl1—Ru1—Cl2	90.66 (2)	C5—C4—H4	120.7 (16)
C8—N1—C2	112.4 (2)	C3—C4—H4	119.8 (16)
C8—N1—C1	107.8 (2)	C6—C5—C4	118.7 (2)
C2—N1—C1	106.6 (2)	C6—C5—H5	120.2 (17)
C8—N1—Ru1	106.67 (15)	C4—C5—H5	120.8 (16)
C2—N1—Ru1	106.15 (13)	C5—C6—C7	119.3 (2)
C1—N1—Ru1	117.32 (15)	C5—C6—H6	122.0 (17)
C7—N2—C3	118.54 (19)	C7—C6—H6	118.6 (17)
C7—N2—Ru1	126.34 (16)	N2—C7—C6	122.2 (2)
C3—N2—Ru1	113.84 (14)	N2—C7—H7	115.1 (16)
C13—N3—C9	118.5 (2)	C6—C7—H7	122.7 (16)
C13—N3—Ru1	126.20 (16)	N1—C8—C9	113.56 (19)
C9—N3—Ru1	114.71 (16)	N1—C8—H8A	108.0 (18)
O1—S1—C14	105.03 (13)	C9—C8—H8A	106.0 (18)
O1—S1—C15	106.66 (13)	N1—C8—H8B	111.6 (18)
C14—S1—C15	99.25 (15)	C9—C8—H8B	109.2 (18)
O1—S1—Ru1	120.38 (8)	H8A—C8—H8B	108 (2)
C14—S1—Ru1	110.28 (9)	N3—C9—C10	121.1 (2)
C15—S1—Ru1	112.91 (11)	N3—C9—C8	115.5 (2)
S1—C14—H14A	112 (2)	C10—C9—C8	123.2 (2)
S1—C14—H14B	108.9 (19)	C11—C10—C9	119.8 (2)
H14A—C14—H14B	108 (3)	C11—C10—H10	121.3 (18)
S1—C14—H14C	105.6 (19)	C9—C10—H10	118.9 (18)
H14A—C14—H14C	112 (3)	C10—C11—C12	119.3 (2)
H14B—C14—H14C	111 (3)	C10—C11—H11	124 (2)
S1—C15—H15A	108.2 (18)	C12—C11—H11	115 (2)
S1—C15—H15B	107 (2)	C13—C12—C11	118.4 (3)
H15A—C15—H15B	115 (3)	C13—C12—H12	123.9 (16)
S1—C15—H15C	112.1 (17)	C11—C12—H12	117.6 (16)
H15A—C15—H15C	111 (2)	N3—C13—C12	122.8 (2)
H15B—C15—H15C	104 (3)	N3—C13—H13	113 (2)
N1—C1—H1A	111.2 (14)	C12—C13—H13	124 (2)
N1—C1—H1B	110.4 (18)		
			
N1—C8—C9—N3	−28.0 (3)	N1—C2—C3—N2	34.3 (3)

### Hydrogen-bond geometry (Å, º)

**Table:**

*D*—H···*A*	*D*—H	H···*A*	*D*···*A*	*D*—H···*A*
C14—H14*A*···O1^i^	0.91 (4)	2.54 (4)	3.431 (3)	169 (3)
C4—H4···Cl1^ii^	0.93 (3)	2.58 (3)	3.487 (2)	165 (2)
C1—H1*C*···O1	0.99 (3)	2.32 (3)	3.182 (4)	145 (2)

Symmetry codes: (i) −*x*+1, −*y*+1, −*z*+1; (ii) *x*+1/2, *y*−1/2, *z*.
