# Supplementary material for: Crystal structure of cis,fac-{N,N-bis­[(pyridin-2-yl)meth­yl]methyl­amine-κ3 N,N′,N′′}di­chlorido­(dimethyl sulfoxide-κS)ruthenium(II)
Source: Acta Crystallogr E Crystallogr Commun. 2015 Aug 22;71(Pt 9):m169–70. doi: 10.1107/S2056989015014875 (PMC4555425; doi:10.1107/S2056989015014875)
Supplement: Supplementary file 3 [file e-71-0m169-sup3.docx]

**Supporting Information**

| **Crystal Structure of *cis,fac*-Dichlorido[*N*,*N*-bis( 2-pyridylmethyl)methylamine](dimethyl sulfoxide)ruthenium(II)** |
| --- |

| Elliott Hulley,* Kasey Trotter and Navamoney Arulsamy  1000 E University Ave, Dept. 3838, Laramie, WY 82071, United States |
| --- |

| [Correspondence e-mail: ehulley@uwyo.edu](SIMON%20_publ_contact_author_email) |
| --- |

**S1. Comment**

Ruthenium(II) complexes of pyridine-based ligands which also contain a dimethyl sulfoxide (dmso) ligand act as catalytic initiators (Bressan & Morvillo 1992; Carvalho *et al.*, 2014; Ferrer *et al.*, 2013). The ambidentate dmso appears to show preferential binding through its sulfur atom with Ru(II) centers, and its O atom with Ru(III) centers (Roeser *et al.*, 2013; Smith *et al.*, 2000). Ruthenium(II) complexes containing the labile dmso and chloride ligands are particularly attractive precursors for the synthesis of specifically-designed catalysts. Our research project is aimed at the catalytic reduction of stable anions such as perchlorates using Ru(II) precatalysts. Multidentate ligands are expected to stabilize ruthenium(IV)-oxido intermediates suggested as intermediates in the catalytic oxidation of a variety of organic substrates in the presence of hypochlorite, perchlorate and other oxidizers (Bressan & Morvillo 1992; Holm, 1987). Here we wish to report the X-ray crystal structural determination of a potential precursor ruthenium complex. The title compound, RuCl_2_(bpma)(dmso), is synthesized from the reaction of RuCl_2_(dmso)_4_ (Evans *et al.,* 1973 ) with *N*,*N*-bis(2-pyridylmethyl)methylamine (bpma) (Astner *et al.,* 2008).

**S2. Structural commentary**

The asymmetric unit contains a well-ordered RuCl_2_(bpma)(dmso) molecule. The metal center is in a distorted-octahedral geometry with the tridentate bpma ligand binding through its two pyridyl N atoms and aliphatic N atom in a facial mode as shown in Fig. 1. The two chloride ligands occupy two adjacent sites, and the dmso ligand is present trans to one of the pyridyl N atoms. The tridentate ligand is folded to achieve facial coordination, and the extent of folding is reflected in the small dihedral angle of 64.55(8)° between the mean planes passing through the two pyridine rings. The two N-Ru-N bite angles of the ligand at 81.70(7) and 82.34(8)° are illustrative of the distorted octahedral geometry of the metal center. The complex can be represented as the *cis,fac*-isomer to indicate the cis-geometry of the dmso ligand to the aliphatic N atom and the facial coordination mode of bpma. A literature survey of Ru(II) complexes of bpma and those of closely related bis(2-pyridylmethyl)alkylamine ligands reveals that an overwhelming majority of the complexes contain facially coordinated tridentate ligands (Dakkach *et al*., 2013; Fisher *et al*., 2009; Mishra *et al*., 2009; Matsuya *et al*., 2009; Mola *et al*., 2009; Mola *et al*., 2006; Mola *et al*., 2007; Rodriguez *et al*., 2001; Sala *et al*., 2008; Serrano *et al*., 2006; Shimuzu *et al*., 2008; Suzuki *et al*., 2014). The *cis,fac*-isomer is the thermodynamically favored (Mola *et al*., 2007), and therefore the more frequent occurrence of this isomer is unsurprising. However, Shimuzu *et al* suggest that the binding mode of the tridentate ligand depends on the nature of the other ligands with the hydroxo and methoxo ligands favoring meridional coordination mode for the tridentate ligands (Shimuzu et al, 2008).

The Ru–N_py_ distances in the present complex are unequal as they have either a chloride or dmso ligands in their respective trans positions. The Ru–S_dmso_ bond is unexceptional at 2.2207(6) Å, and comparable to those found in cis,fac-RuCl_2_(bpma)(dmso) and tans,mer-RuCl_2_(bpea)(dmso) (Mola *et al*., 2007).

**Figure 1.** View of RuCl_2_(dpma)(dmso). Hydrogen atoms are omitted, and the thermal parameters are drawn at 50% probability.

**S3. References**

Astner, J., Weitzer, M., Foxon, S. P., Schindler, S., Heinemann, F. W., Mukherjee, J., Gupta, R., Mahadevan, V. & Mukherjee, R. (2008). *Inorg. Chem. Acta*, **341 (1)**, 279 – 292.

Bressan, M. & Morvillo, A. (1992). *J. Mol. Catal***. 71 (2)**, 149 – 155.

Carvalho, V. P., Ferraz, C. P. & Lima-Neto, B. S. (2014). *Inorg. Chim. Acta* **418**, 1–7.

Dakkach, M., Atlamsani, A., Parella, T., Fontrodona, X., Romero, I. & Rodriguez, M. (2013). *Inorg. Chem.* **52 (9)**, 5077 – 5087.

Evans, I. P., Spencer, A. & Wilkinson, G. (1973). *J. Chem. Soc., Dalton Trans.* **(2)**, 204 – 209.

Ferrer, I., Rich, J., Fontrodona, X., Rodriquez, M. & Romero, I. (2013). *Dalton Trans.* **42 (2)**, 13461 – 13469.

Fischer, P. J., Minasian, S. G. & Arnold, J. (2009). *Acta Cryst.* **E65**, m1371 – 1372.

Mishra, H., Patra, A. K. & Mukherjee, R. (2009). *Inorg. Chim. Acta* **362 (2)**, 483 – 490.

Matsuya, K., Fukui, S., Hoshino, Y. & Nagao, H. (2009). *Dalton Trans.* **(38)**, 7876 – 7878.

Mola, J., Pujol, D., Rodriguez, M., Romero, I., Sala, X., Katz, N., Parella, T., Benet-Buchholz, J., Fontrodona, X. & Llobet, A. (2009). *Aust. J. Chem.* **62 (12)**, 1675 – 1683.

Mola, J., Rodriguez, M., Romero, I., Llobet, A., Parella, T., Poater, A., Duran, M., Sola, M. & Benet-Buchholz, J. (2006). *Inorg. Chem.* **45 (26)**, 10520 – 10529.

Mola, J., Romero, I., Rodriguez, M., Bozoglian, F., Poater, A., Sola, M., Parella, T., Benet-Buchholz, J., Fontrodona, X. & Llobet, A. (2007). *Inorg. Chem.* **46 (25)**, 10707 – 10716.

Rodriguez, M., Romero, I., Llobet, A., Deronzier, A., Biner, M., Parella, T. & Stoeckli-Evans, H. (2001). *Inorg. Chem.* **40 (17)**, 4150 -4156.

Roeser, S., Maji, S., Benet-Buchholz, J., Pons, J. & Llobet, A. (2013). *Eur. J. Inorg. Chem.* **(2)**, 232 – 240.

Sala, X., Poater, A., von Zelewsky, A., Parella, T., Fontrodona, X., Romero, I., Sola, M., Rodriguez, M. & Llobet, A. (2008). *Inorg. Chem.* **47 (18)**, 8016 – 8024.

Serrano, I., Rodriguez, M., Romero, I., Llobet, A., Parella, T., Campelo, J. M., Luna, D., Marinas, J. M. & Benet-Buchholz, J. (2006). *Inorg. Chem.* **45 (6)**, 2644 – 2651.

Shimizu, Y., Fukui, S., Oi, T. & Nagao, H. (2008). *Bull. Chem. Soc. Jpn.* **81 (10)**, 1285 – 1295.

Smith, M. K., Gibson, J. A., Young, C. G., Broomhead, J. A., Junk, P. C. & Keene, F. R. (2000). *Eur. J. Inorg. Chem.* **(6)**, 1365 – 1370.

Suzuki, T., Matsuya, K., Kawamoto, T. & Nagao, H. (2014). *Eur. J. Inorg. Chem.* **(4)**, 722 – 727.

**Table S1.** Crystal data and structure refinement for RuCl_2_(dpma)(dmso).

Identification code KNT02

Empirical formula C15 H21 Cl2 N3 O Ru S

Formula weight 463.38

Temperature 150(2) K

Wavelength 0.71073 Å

Crystal system Monoclinic

Space group C2/c

Unit cell dimensions a = 14.6117(3) Å α = 90°.

b = 9.3345(2) Å β = 102.734(1)°.

c = 27.3451(7) Å γ = 90°.

Volume 3637.94(14) Å3

Z 8

Density (calculated) 1.692 Mg/m3

Absorption coefficient 1.277 mm-1

F(000) 1872

Crystal size 0.214 × 0.165 × 0.114 mm3

Theta range for data collection 2.608 to 33.726°.

Index ranges -19 ≤ *h* ≤ 22, -14 ≤ *k* ≤ 14, -42 ≤ *l* ≤ 42

Reflections collected 33234

Independent reflections 7273 [R(int) = 0.0678]

Completeness to theta = 25.242° 99.8 %

Absorption correction Semi-empirical from equivalents

Max. and min. transmission 0.7469 and 0.6471

Refinement method Full-matrix least-squares on F2

Data / restraints / parameters 7273 / 0 / 292

Goodness-of-fit on F2 1.008

Final R indices [I>2sigma(I)] R1 = 0.0389, wR2 = 0.0748

R indices (all data) R1 = 0.0660, wR2 = 0.0847

Extinction coefficient n/a

Largest diff. peak and hole 1.125 and -0.916 e.Å-3

**Table S2.** Atomic coordinates (× 104) and equivalent isotropic displacement parameters (Å2 × 103)

for RuCl_2_(dpma)(dmso). U(eq) is defined as one third of the trace of the orthogonalized Uij tensor.

________________________________________________________________________________

x y z U(eq)

________________________________________________________________________________

Ru(1) 6734(1) 5114(1) 3764(1) 16(1)

Cl(1) 5445(1) 6528(1) 3300(1) 20(1)

Cl(2) 7705(1) 7252(1) 3946(1) 26(1)

N(1) 7899(1) 3807(2) 4088(1) 22(1)

N(2) 7232(1) 4714(2) 3114(1) 17(1)

N(3) 6103(1) 3164(2) 3584(1) 20(1)

S(1) 6116(1) 5302(1) 4434(1) 24(1)

O(1) 6494(2) 4440(2) 4890(1) 39(1)

C(14) 4890(2) 4893(3) 4272(1) 32(1)

C(15) 6052(2) 7116(3) 4638(1) 35(1)

C(1) 8474(2) 4251(3) 4589(1) 31(1)

C(2) 8541(2) 3835(3) 3731(1) 24(1)

C(3) 8027(2) 3934(2) 3194(1) 18(1)

C(4) 8361(2) 3340(2) 2803(1) 22(1)

C(5) 7877(2) 3565(2) 2316(1) 23(1)

C(6) 7067(2) 4370(3) 2233(1) 22(1)

C(7) 6758(2) 4919(2) 2639(1) 19(1)

C(8) 7522(2) 2349(3) 4143(1) 29(1)

C(9) 6644(2) 2018(2) 3763(1) 23(1)

C(10) 6356(2) 633(3) 3621(1) 32(1)

C(11) 5508(2) 411(3) 3303(1) 36(1)

C(12) 4947(2) 1582(3) 3123(1) 32(1)

C(13) 5280(2) 2934(3) 3266(1) 24(1)

________________________________________________________________________________ **Table S3.** Bond lengths [Å] and angles [°] for RuCl_2_(dpma)(dmso).

_____________________________________________________

Ru(1)-N(3) 2.0515(19)

Ru(1)-N(2) 2.0989(18)

Ru(1)-N(1) 2.1224(19)

Ru(1)-S(1) 2.2207(6)

Ru(1)-Cl(1) 2.4187(5)

Ru(1)-Cl(2) 2.4352(6)

N(1)-C(8) 1.489(3)

N(1)-C(2) 1.495(3)

N(1)-C(1) 1.499(3)

N(2)-C(7) 1.342(3)

N(2)-C(3) 1.347(3)

N(3)-C(13) 1.339(3)

N(3)-C(9) 1.355(3)

S(1)-O(1) 1.4838(18)

S(1)-C(14) 1.788(3)

S(1)-C(15) 1.791(3)

C(14)-H(14A) 0.91(4)

C(14)-H(14B) 0.96(3)

C(14)-H(14C) 0.96(3)

C(15)-H(15A) 0.97(3)

C(15)-H(15B) 1.03(4)

C(15)-H(15C) 0.97(3)

C(1)-H(1A) 0.99(3)

C(1)-H(1B) 0.99(3)

C(1)-H(1C) 0.99(3)

C(2)-C(3) 1.497(3)

C(2)-H(2A) 0.99(3)

C(2)-H(2B) 1.02(3)

C(3)-C(4) 1.387(3)

C(4)-C(5) 1.378(3)

C(4)-H(4) 0.93(3)

C(5)-C(6) 1.378(3)

C(5)-H(5) 0.90(2)

C(6)-C(7) 1.385(3)

C(6)-H(6) 0.92(3)

C(7)-H(7) 0.97(3)

C(8)-C(9) 1.493(4)

C(8)-H(8A) 0.98(3)

C(8)-H(8B) 0.89(3)

C(9)-C(10) 1.389(3)

C(10)-C(11) 1.364(4)

C(10)-H(10) 0.92(3)

C(11)-C(12) 1.390(4)

C(11)-H(11) 0.90(3)

C(12)-C(13) 1.378(3)

C(12)-H(12) 0.97(3)

C(13)-H(13) 0.85(3)

N(3)-Ru(1)-N(2) 81.96(7)

N(3)-Ru(1)-N(1) 82.34(8)

N(2)-Ru(1)-N(1) 81.70(7)

N(3)-Ru(1)-S(1) 91.40(5)

N(2)-Ru(1)-S(1) 173.35(5)

N(1)-Ru(1)-S(1) 97.82(5)

N(3)-Ru(1)-Cl(1) 95.80(5)

N(2)-Ru(1)-Cl(1) 91.57(5)

N(1)-Ru(1)-Cl(1) 173.20(5)

S(1)-Ru(1)-Cl(1) 88.75(2)

N(3)-Ru(1)-Cl(2) 170.84(6)

N(2)-Ru(1)-Cl(2) 91.40(5)

N(1)-Ru(1)-Cl(2) 90.49(6)

S(1)-Ru(1)-Cl(2) 95.24(2)

Cl(1)-Ru(1)-Cl(2) 90.66(2)

C(8)-N(1)-C(2) 112.4(2)

C(8)-N(1)-C(1) 107.8(2)

C(2)-N(1)-C(1) 106.6(2)

C(8)-N(1)-Ru(1) 106.67(15)

C(2)-N(1)-Ru(1) 106.15(13)

C(1)-N(1)-Ru(1) 117.32(15)

C(7)-N(2)-C(3) 118.54(19)

C(7)-N(2)-Ru(1) 126.34(16)

C(3)-N(2)-Ru(1) 113.84(14)

C(13)-N(3)-C(9) 118.5(2)

C(13)-N(3)-Ru(1) 126.20(16)

C(9)-N(3)-Ru(1) 114.71(16)

O(1)-S(1)-C(14) 105.03(13)

O(1)-S(1)-C(15) 106.66(13)

C(14)-S(1)-C(15) 99.25(15)

O(1)-S(1)-Ru(1) 120.38(8)

C(14)-S(1)-Ru(1) 110.28(9)

C(15)-S(1)-Ru(1) 112.91(11)

S(1)-C(14)-H(14A) 112(2)

S(1)-C(14)-H(14B) 108.9(19)

H(14A)-C(14)-H(14B) 108(3)

S(1)-C(14)-H(14C) 105.6(19)

H(14A)-C(14)-H(14C) 112(3)

H(14B)-C(14)-H(14C) 111(3)

S(1)-C(15)-H(15A) 108.2(18)

S(1)-C(15)-H(15B) 107(2)

H(15A)-C(15)-H(15B) 115(3)

S(1)-C(15)-H(15C) 112.1(17)

H(15A)-C(15)-H(15C) 111(2)

H(15B)-C(15)-H(15C) 104(3)

N(1)-C(1)-H(1A) 111.2(14)

N(1)-C(1)-H(1B) 110.4(18)

H(1A)-C(1)-H(1B) 110(2)

N(1)-C(1)-H(1C) 107.1(17)

H(1A)-C(1)-H(1C) 109(2)

H(1B)-C(1)-H(1C) 109(2)

N(1)-C(2)-C(3) 112.92(19)

N(1)-C(2)-H(2A) 111.3(15)

C(3)-C(2)-H(2A) 113.0(15)

N(1)-C(2)-H(2B) 104.0(17)

C(3)-C(2)-H(2B) 107.0(17)

H(2A)-C(2)-H(2B) 108(2)

N(2)-C(3)-C(4) 121.8(2)

N(2)-C(3)-C(2) 114.98(19)

C(4)-C(3)-C(2) 123.2(2)

C(5)-C(4)-C(3) 119.5(2)

C(5)-C(4)-H(4) 120.7(16)

C(3)-C(4)-H(4) 119.8(16)

C(6)-C(5)-C(4) 118.7(2)

C(6)-C(5)-H(5) 120.2(17)

C(4)-C(5)-H(5) 120.8(16)

C(5)-C(6)-C(7) 119.3(2)

C(5)-C(6)-H(6) 122.0(17)

C(7)-C(6)-H(6) 118.6(17)

N(2)-C(7)-C(6) 122.2(2)

N(2)-C(7)-H(7) 115.1(16)

C(6)-C(7)-H(7) 122.7(16)

N(1)-C(8)-C(9) 113.56(19)

N(1)-C(8)-H(8A) 108.0(18)

C(9)-C(8)-H(8A) 106.0(18)

N(1)-C(8)-H(8B) 111.6(18)

C(9)-C(8)-H(8B) 109.2(18)

H(8A)-C(8)-H(8B) 108(2)

N(3)-C(9)-C(10) 121.1(2)

N(3)-C(9)-C(8) 115.5(2)

C(10)-C(9)-C(8) 123.2(2)

C(11)-C(10)-C(9) 119.8(2)

C(11)-C(10)-H(10) 121.3(18)

C(9)-C(10)-H(10) 118.9(18)

C(10)-C(11)-C(12) 119.3(2)

C(10)-C(11)-H(11) 124(2)

C(12)-C(11)-H(11) 115(2)

C(13)-C(12)-C(11) 118.4(3)

C(13)-C(12)-H(12) 123.9(16)

C(11)-C(12)-H(12) 117.6(16)

N(3)-C(13)-C(12) 122.8(2)

N(3)-C(13)-H(13) 113(2)

C(12)-C(13)-H(13) 124(2)

_____________________________________________________________

**Table S4.** Anisotropic displacement parameters (Å2 × 103) for RuCl_2_(dpma)(dmso). The anisotropic

displacement factor exponent takes the form: -2π2[ h2 a*2U11 + ... + 2 h k a* b* U12 ]

______________________________________________________________________________

U11 U22 U33 U23 U13 U12

______________________________________________________________________________

Ru(1) 19(1) 17(1) 14(1) 2(1) 4(1) 5(1)

Cl(1) 20(1) 19(1) 21(1) 4(1) 5(1) 7(1)

Cl(2) 29(1) 23(1) 25(1) -1(1) 0(1) 0(1)

N(1) 23(1) 25(1) 19(1) 3(1) 5(1) 9(1)

N(2) 19(1) 16(1) 16(1) 1(1) 5(1) 3(1)

N(3) 24(1) 18(1) 19(1) 3(1) 10(1) 5(1)

S(1) 30(1) 28(1) 16(1) 4(1) 9(1) 12(1)

O(1) 50(1) 51(1) 21(1) 16(1) 17(1) 27(1)

C(14) 35(2) 39(2) 27(1) 5(1) 18(1) 8(1)

C(15) 43(2) 34(1) 29(1) -7(1) 12(1) 10(1)

C(1) 30(2) 41(2) 18(1) 1(1) -2(1) 10(1)

C(2) 17(1) 32(1) 22(1) 2(1) 4(1) 8(1)

C(3) 18(1) 18(1) 18(1) 2(1) 4(1) 1(1)

C(4) 20(1) 24(1) 23(1) -2(1) 7(1) 4(1)

C(5) 24(1) 25(1) 22(1) -5(1) 9(1) 0(1)

C(6) 23(1) 26(1) 17(1) 1(1) 5(1) 1(1)

C(7) 19(1) 22(1) 16(1) 0(1) 4(1) 1(1)

C(8) 31(2) 25(1) 32(1) 11(1) 7(1) 11(1)

C(9) 27(1) 20(1) 25(1) 6(1) 11(1) 6(1)

C(10) 38(2) 20(1) 42(2) 6(1) 21(1) 8(1)

C(11) 41(2) 20(1) 51(2) -5(1) 22(1) -6(1)

C(12) 30(2) 25(1) 41(2) -4(1) 10(1) -6(1)

C(13) 24(1) 22(1) 28(1) 2(1) 9(1) 2(1)

______________________________________________________________________________ **Table S5.** Hydrogen coordinates (× 104) and isotropic displacement parameters (Å2 × 103)

for RuCl_2_(dpma)(dmso).

________________________________________________________________________________

x y z U(eq)

________________________________________________________________________________

H(14A) 4590(30) 5150(30) 4517(13) 45(10)

H(14B) 4610(20) 5410(30) 3971(12) 38(8)

H(14C) 4850(20) 3880(40) 4213(11) 42(9)

H(15A) 5710(20) 7130(30) 4902(11) 37(8)

H(15B) 6730(30) 7480(40) 4742(12) 63(11)

H(15C) 5760(20) 7750(30) 4363(10) 33(8)

H(1A) 8987(18) 3560(30) 4712(9) 18(6)

H(1B) 8730(20) 5230(30) 4570(11) 33(8)

H(1C) 8050(20) 4270(30) 4826(10) 28(7)

H(2A) 8994(19) 3030(30) 3795(9) 27(7)

H(2B) 8910(20) 4770(30) 3815(11) 30(8)

H(4) 8920(20) 2830(30) 2870(9) 25(7)

H(5) 8051(18) 3120(30) 2059(9) 22(7)

H(6) 6718(19) 4550(30) 1914(10) 24(7)

H(7) 6170(20) 5450(30) 2603(10) 27(7)

H(8A) 7360(20) 2300(30) 4470(11) 41(9)

H(8B) 7950(20) 1670(30) 4131(10) 27(7)

H(10) 6740(20) -120(30) 3745(10) 28(8)

H(11) 5320(20) -430(40) 3154(12) 46(9)

H(12) 4356(19) 1400(30) 2891(10) 24(7)

H(13) 4990(20) 3700(30) 3156(10) 29(7)

________________________________________________________________________________ **Table S6.** Torsion angles [°] for RuCl_2_(dpma)(dmso).

________________________________________________________________

N(1)-C(8)-C(9)-N(3) -28.0(3)

N(1)-C(2)-C(3)-N(2) 34.3(3)

________________________________________________________________
