# Supplementary material for: Crystal structure of cis,fac-{N,N-bis­[(pyridin-2-yl)meth­yl]methyl­amine-κ3 N,N′,N′′}di­chlorido­(dimethyl sulfoxide-κS)ruthenium(II)
Source: Acta Crystallogr E Crystallogr Commun. 2015 Aug 22;71(Pt 9):m169–70. doi: 10.1107/S2056989015014875 (PMC4555425; doi:10.1107/S2056989015014875)
Supplement: Supplementary file 4 [file e-71-0m169-sup3.pdf]

Crystal Structure of *cis,fac*-Dichlorido[*N,N*-bis(2-pyridylmethyl)methylamine](dimethyl sulfoxide)ruthenium(II)

Elliott Hulley,* Kasey Trotter and Navamoney Arulsamy

1000 E University Ave, Dept. 3838, Laramie, WY 82071, United States

Correspondence e-mail: ehulley@uwyo.edu

S1. Comment

Ruthenium(II) complexes of pyridine-based ligands which also contain a dimethyl sulfoxide (dmso) ligand act as catalytic initiators (Bressan & Morvillo 1992; Carvalho *et al.*, 2014; Ferrer *et al.*, 2013). The ambidentate dmso appears to show preferential binding through its sulfur atom with Ru(II) centers, and its O atom with Ru(III) centers (Roeser *et al.*, 2013; Smith *et al.*, 2000). Ruthenium(II) complexes containing the labile dmso and chloride ligands are particularly attractive precursors for the synthesis of specifically-designed catalysts. Our research project is aimed at the catalytic reduction of stable anions such as perchlorates using Ru(II) precatalysts. Multidentate ligands are expected to stabilize ruthenium(IV)-oxido intermediates suggested as intermediates in the catalytic oxidation of a variety of organic substrates in the presence of hypochlorite, perchlorate and other oxidizers (Bressan & Morvillo 1992; Holm, 1987). Here we wish to report the X-ray crystal structural determination of a potential precursor ruthenium complex. The title compound, $RuCl_2$(bpma)(dmso), is synthesized from the reaction of $RuCl_2(dmso)_4$ (Evans *et al.*, 1973) with *N*,*N*-bis(2-pyridylmethyl)methylamine (bpma) (Astner *et al.*, 2008).

S2. Structural commentary

The asymmetric unit contains a well-ordered $RuCl_2$(bpma)(dmso) molecule. The metal center is in a distorted-octahedral geometry with the tridentate bpma ligand binding through its two pyridyl N atoms and aliphatic N atom in a facial mode as shown in Fig. 1. The two chloride ligands occupy two adjacent sites, and the dmso ligand is present trans to one of the pyridyl N atoms. The tridentate ligand is folded to achieve facial coordination, and the extent of folding is reflected in the small dihedral angle of 64.55(8)° between the mean planes passing through the two pyridine rings. The two N-Ru-N bite angles of the ligand at 81.70(7) and 82.34(8)° are illustrative of the distorted octahedral geometry of the metal center. The complex can be represented as the *cis*,*fac*-isomer to indicate the cis-geometry of the dmso ligand to the aliphatic N atom and the facial coordination mode of bpma. A literature survey of Ru(II) complexes of bpma and those of closely related bis(2-pyridylmethyl)alkylamine ligands reveals that an overwhelming majority of the complexes contain facially coordinated tridentate ligands (Dakkach *et al*., 2013; Fisher *et al*., 2009; Mishra *et al*., 2009; Matsuya *et al*., 2009; Mola *et al*., 2009; Mola *et al*., 2006; Mola *et al*., 2007; Rodriguez *et al*., 2001; Sala *et al*., 2008; Serrano *et al*., 2006; Shimuzu *et al*., 2008; Suzuki *et al*., 2014). The *cis,fac*-isomer is the thermodynamically favored (Mola *et al*., 2007), and therefore the more frequent occurrence of this isomer is unsurprising. However, Shimuzu *et al* suggest that the binding mode of the tridentate ligand depends on the nature of the other ligands with the hydroxo and methoxo ligands favoring meridional coordination mode for the tridentate ligands (Shimuzu et al, 2008).

The Ru–N_{py} distances in the present complex are unequal as they have either a chloride or dmso ligands in their respective trans positions. The Ru–S_{dmso} bond is unexceptional at 2.2207(6) Å, and comparable to those found in cis,fac-$RuCl_2$(bpma)(dmso) and tans,mer-$RuCl_2$(bpea)(dmso) (Mola *et al*., 2007).

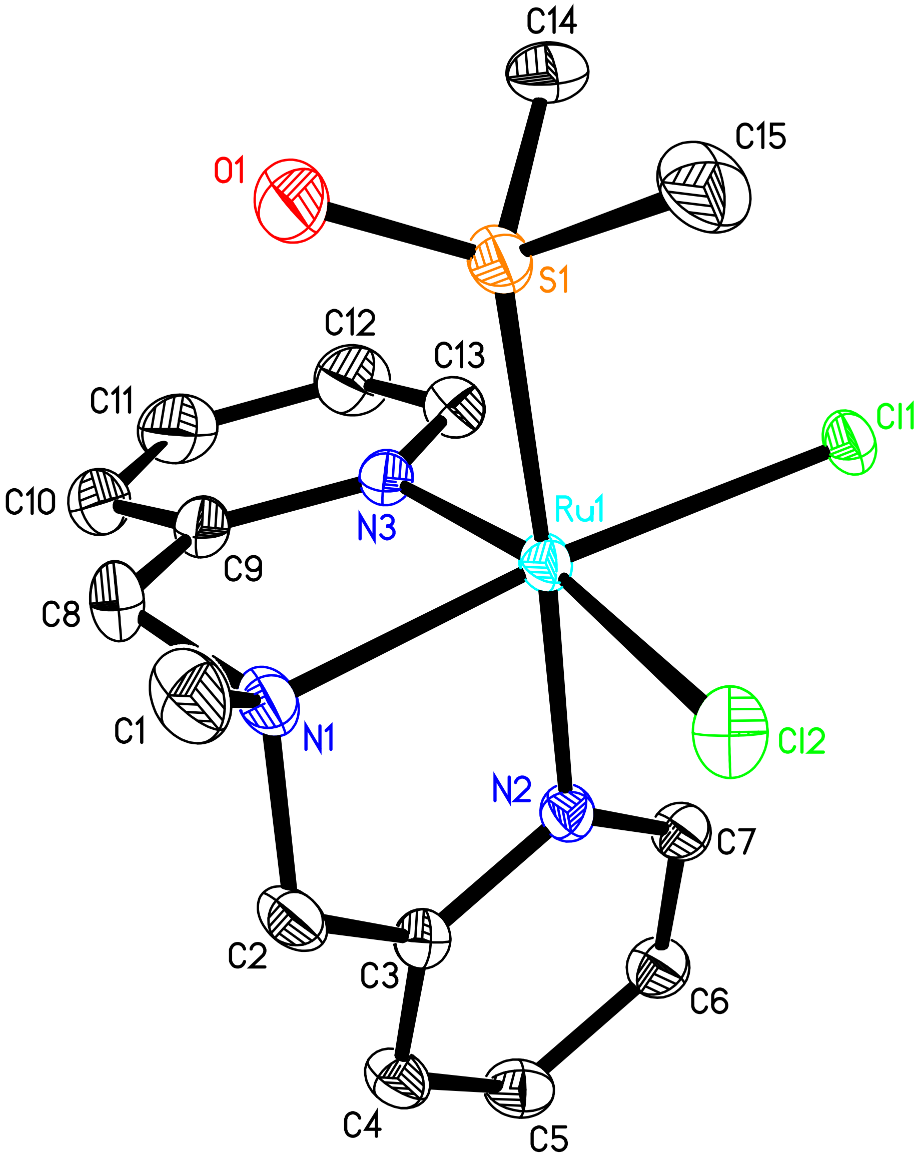

Figure 1. View of $RuCl_2$(dpma)(dmso). Hydrogen atoms are omitted, and the thermal parameters are drawn at 50% probability.

S3. References

Astner, J., Weitzer, M., Foxon, S. P., Schindler, S., Heinemann, F. W., Mukherjee, J., Gupta, R., Mahadevan, V. & Mukherjee, R. (2008). *Inorg. Chem. Acta*, **341 (1)**, 279 – 292.

Bressan, M. & Morvillo, A. (1992). *J. Mol. Catal*. **71 (2)**, 149 – 155.

Carvalho, V. P., Ferraz, C. P. & Lima-Neto, B. S. (2014). *Inorg. Chim. Acta* **418**, 1–7.

Dakkach, M., Atlamsani, A., Parella, T., Fontrodona, X., Romero, I. & Rodriguez, M. (2013). *Inorg. Chem.* **52 (9)**, 5077 – 5087.

Evans, I. P., Spencer, A. & Wilkinson, G. (1973). *J. Chem. Soc., Dalton Trans.* **(2)**, 204 – 209.

Ferrer, I., Rich, J., Fontrodona, X., Rodriquez, M. & Romero, I. (2013). *Dalton Trans.* **42 (2)**, 13461 – 13469.

Fischer, P. J., Minasian, S. G. & Arnold, J. (2009). *Acta Cryst.* **E65**, m1371 – 1372.

Mishra, H., Patra, A. K. & Mukherjee, R. (2009). *Inorg. Chim. Acta* **362 (2)**, 483 – 490.

Matsuya, K., Fukui, S., Hoshino, Y. & Nagao, H. (2009). *Dalton Trans.* **(38)**, 7876 – 7878.

Mola, J., Pujol, D., Rodriguez, M., Romero, I., Sala, X., Katz, N., Parella, T., Benet-Buchholz, J., Fontrodona, X. & Llobet, A. (2009). *Aust. J. Chem.* **62 (12)**, 1675 – 1683.

Mola, J., Rodriguez, M., Romero, I., Llobet, A., Parella, T., Poater, A., Duran, M., Sola, M. & Benet-Buchholz, J. (2006). *Inorg. Chem.* **45 (26)**, 10520 – 10529.

Mola, J., Romero, I., Rodriguez, M., Bozoglian, F., Poater, A., Sola, M., Parella, T., Benet-Buchholz, J., Fontrodona, X. & Llobet, A. (2007). *Inorg. Chem.* **46 (25)**, 10707 – 10716.

Rodriguez, M., Romero, I., Llobet, A., Deronzier, A., Biner, M., Parella, T. & Stoeckli-Evans, H. (2001). *Inorg. Chem.* **40 (17)**, 4150 -4156.

Roeser, S., Maji, S., Benet-Buchholz, J., Pons, J. & Llobet, A. (2013). *Eur. J. Inorg. Chem.* **(2)**, 232 – 240.

Sala, X., Poater, A., von Zelewsky, A., Parella, T., Fontrodona, X., Romero, I., Sola, M., Rodriguez, M. & Llobet, A. (2008). *Inorg. Chem.* **47 (18)**, 8016 – 8024.

Serrano, I., Rodriguez, M., Romero, I., Llobet, A., Parella, T., Campelo, J. M., Luna, D., Marinas, J. M. & Benet-Buchholz, J. (2006). *Inorg. Chem.* **45 (6)**, 2644 – 2651.

Shimizu, Y., Fukui, S., Oi, T. & Nagao, H. (2008). *Bull. Chem. Soc. Jpn.* **81 (10)**, 1285 – 1295.

Smith, M. K., Gibson, J. A., Young, C. G., Broomhead, J. A., Junk, P. C. & Keene, F. R. (2000). *Eur. J. Inorg. Chem.* **(6)**, 1365 – 1370.

Suzuki, T., Matsuya, K., Kawamoto, T. & Nagao, H. (2014). *Eur. J. Inorg. Chem.* **(4)**, 722 – 727.

Table S1. Crystal data and structure refinement for $RuCl_2$(dpma)(dmso).

Identification code	KNT02	
Empirical formula	C15 H21 Cl2 N3 O Ru S	
Formula weight	463.38	
Temperature	150(2) K	
Wavelength	0.71073 Å	
Crystal system	Monoclinic	
Space group	C2/c	
Unit cell dimensions	a = 14.6117(3) Å	α = 90°.
	b = 9.3345(2) Å	β = 102.734(1)°.
	c = 27.3451(7) Å	γ = 90°.
Volume	3637.94(14) Å^3	
Z	8	
Density (calculated)	1.692 Mg/m^3	
Absorption coefficient	1.277 mm^{-1}	
F(000)	1872	
Crystal size	0.214 × 0.165 × 0.114 mm^3	
Theta range for data collection	2.608 to 33.726°.	
Index ranges	$-19 \leq h \leq 22$, $-14 \leq k \leq 14$, $-42 \leq l \leq 42$	
Reflections collected	33234	
Independent reflections	7273 [R(int) = 0.0678]	
Completeness to theta = 25.242°	99.8 %	
Absorption correction	Semi-empirical from equivalents	
Max. and min. transmission	0.7469 and 0.6471	
Refinement method	Full-matrix least-squares on F^2	
Data / restraints / parameters	7273 / 0 / 292	
Goodness-of-fit on F^2	1.008	
Final R indices [I>2sigma(I)]	R1 = 0.0389, wR2 = 0.0748	
R indices (all data)	R1 = 0.0660, wR2 = 0.0847	
Extinction coefficient	n/a	
Largest diff. peak and hole	1.125 and -0.916 e.Å^{-3}	

Table S2. Atomic coordinates (× 10^4) and equivalent isotropic displacement parameters (Å^2 × 10^3) for $RuCl_2$(dpma)(dmso). U(eq) is defined as one third of the trace of the orthogonalized U^{ij} tensor.

	x	y	z	U(eq)
Ru(1)	6734(1)	5114(1)	3764(1)	16(1)
Cl(1)	5445(1)	6528(1)	3300(1)	20(1)
Cl(2)	7705(1)	7252(1)	3946(1)	26(1)
N(1)	7899(1)	3807(2)	4088(1)	22(1)
N(2)	7232(1)	4714(2)	3114(1)	17(1)
N(3)	6103(1)	3164(2)	3584(1)	20(1)
S(1)	6116(1)	5302(1)	4434(1)	24(1)
O(1)	6494(2)	4440(2)	4890(1)	39(1)
C(14)	4890(2)	4893(3)	4272(1)	32(1)
C(15)	6052(2)	7116(3)	4638(1)	35(1)
C(1)	8474(2)	4251(3)	4589(1)	31(1)
C(2)	8541(2)	3835(3)	3731(1)	24(1)
C(3)	8027(2)	3934(2)	3194(1)	18(1)
C(4)	8361(2)	3340(2)	2803(1)	22(1)
C(5)	7877(2)	3565(2)	2316(1)	23(1)
C(6)	7067(2)	4370(3)	2233(1)	22(1)
C(7)	6758(2)	4919(2)	2639(1)	19(1)
C(8)	7522(2)	2349(3)	4143(1)	29(1)
C(9)	6644(2)	2018(2)	3763(1)	23(1)
C(10)	6356(2)	633(3)	3621(1)	32(1)
C(11)	5508(2)	411(3)	3303(1)	36(1)
C(12)	4947(2)	1582(3)	3123(1)	32(1)
C(13)	5280(2)	2934(3)	3266(1)	24(1)

Table S3. Bond lengths [Å] and angles [°] for $RuCl_2$(dpma)(dmso).

Ru(1)-N(3)	2.0515(19)
Ru(1)-N(2)	2.0989(18)
Ru(1)-N(1)	2.1224(19)
Ru(1)-S(1)	2.2207(6)
Ru(1)-Cl(1)	2.4187(5)
Ru(1)-Cl(2)	2.4352(6)
N(1)-C(8)	1.489(3)
N(1)-C(2)	1.495(3)
N(1)-C(1)	1.499(3)
N(2)-C(7)	1.342(3)
N(2)-C(3)	1.347(3)
N(3)-C(13)	1.339(3)
N(3)-C(9)	1.355(3)
S(1)-O(1)	1.4838(18)
S(1)-C(14)	1.788(3)
S(1)-C(15)	1.791(3)
C(14)-H(14A)	0.91(4)
C(14)-H(14B)	0.96(3)
C(14)-H(14C)	0.96(3)
C(15)-H(15A)	0.97(3)
C(15)-H(15B)	1.03(4)
C(15)-H(15C)	0.97(3)
C(1)-H(1A)	0.99(3)
C(1)-H(1B)	0.99(3)
C(1)-H(1C)	0.99(3)
C(2)-C(3)	1.497(3)
C(2)-H(2A)	0.99(3)
C(2)-H(2B)	1.02(3)
C(3)-C(4)	1.387(3)
C(4)-C(5)	1.378(3)
C(4)-H(4)	0.93(3)
C(5)-C(6)	1.378(3)
C(5)-H(5)	0.90(2)
C(6)-C(7)	1.385(3)

C(6)-H(6)	0.92(3)
C(7)-H(7)	0.97(3)
C(8)-C(9)	1.493(4)
C(8)-H(8A)	0.98(3)
C(8)-H(8B)	0.89(3)
C(9)-C(10)	1.389(3)
C(10)-C(11)	1.364(4)
C(10)-H(10)	0.92(3)
C(11)-C(12)	1.390(4)
C(11)-H(11)	0.90(3)
C(12)-C(13)	1.378(3)
C(12)-H(12)	0.97(3)
C(13)-H(13)	0.85(3)
N(3)-Ru(1)-N(2)	81.96(7)
N(3)-Ru(1)-N(1)	82.34(8)
N(2)-Ru(1)-N(1)	81.70(7)
N(3)-Ru(1)-S(1)	91.40(5)
N(2)-Ru(1)-S(1)	173.35(5)
N(1)-Ru(1)-S(1)	97.82(5)
N(3)-Ru(1)-Cl(1)	95.80(5)
N(2)-Ru(1)-Cl(1)	91.57(5)
N(1)-Ru(1)-Cl(1)	173.20(5)
S(1)-Ru(1)-Cl(1)	88.75(2)
N(3)-Ru(1)-Cl(2)	170.84(6)
N(2)-Ru(1)-Cl(2)	91.40(5)
N(1)-Ru(1)-Cl(2)	90.49(6)
S(1)-Ru(1)-Cl(2)	95.24(2)
Cl(1)-Ru(1)-Cl(2)	90.66(2)
C(8)-N(1)-C(2)	112.4(2)
C(8)-N(1)-C(1)	107.8(2)
C(2)-N(1)-C(1)	106.6(2)
C(8)-N(1)-Ru(1)	106.67(15)
C(2)-N(1)-Ru(1)	106.15(13)
C(1)-N(1)-Ru(1)	117.32(15)
C(7)-N(2)-C(3)	118.54(19)

C(7)-N(2)-Ru(1)	126.34(16)
C(3)-N(2)-Ru(1)	113.84(14)
C(13)-N(3)-C(9)	118.5(2)
C(13)-N(3)-Ru(1)	126.20(16)
C(9)-N(3)-Ru(1)	114.71(16)
O(1)-S(1)-C(14)	105.03(13)
O(1)-S(1)-C(15)	106.66(13)
C(14)-S(1)-C(15)	99.25(15)
O(1)-S(1)-Ru(1)	120.38(8)
C(14)-S(1)-Ru(1)	110.28(9)
C(15)-S(1)-Ru(1)	112.91(11)
S(1)-C(14)-H(14A)	112(2)
S(1)-C(14)-H(14B)	108.9(19)
H(14A)-C(14)-H(14B)	108(3)
S(1)-C(14)-H(14C)	105.6(19)
H(14A)-C(14)-H(14C)	112(3)
H(14B)-C(14)-H(14C)	111(3)
S(1)-C(15)-H(15A)	108.2(18)
S(1)-C(15)-H(15B)	107(2)
H(15A)-C(15)-H(15B)	115(3)
S(1)-C(15)-H(15C)	112.1(17)
H(15A)-C(15)-H(15C)	111(2)
H(15B)-C(15)-H(15C)	104(3)
N(1)-C(1)-H(1A)	111.2(14)
N(1)-C(1)-H(1B)	110.4(18)
H(1A)-C(1)-H(1B)	110(2)
N(1)-C(1)-H(1C)	107.1(17)
H(1A)-C(1)-H(1C)	109(2)
H(1B)-C(1)-H(1C)	109(2)
N(1)-C(2)-C(3)	112.92(19)
N(1)-C(2)-H(2A)	111.3(15)
C(3)-C(2)-H(2A)	113.0(15)
N(1)-C(2)-H(2B)	104.0(17)
C(3)-C(2)-H(2B)	107.0(17)
H(2A)-C(2)-H(2B)	108(2)
N(2)-C(3)-C(4)	121.8(2)

N(2)-C(3)-C(2)	114.98(19)
C(4)-C(3)-C(2)	123.2(2)
C(5)-C(4)-C(3)	119.5(2)
C(5)-C(4)-H(4)	120.7(16)
C(3)-C(4)-H(4)	119.8(16)
C(6)-C(5)-C(4)	118.7(2)
C(6)-C(5)-H(5)	120.2(17)
C(4)-C(5)-H(5)	120.8(16)
C(5)-C(6)-C(7)	119.3(2)
C(5)-C(6)-H(6)	122.0(17)
C(7)-C(6)-H(6)	118.6(17)
N(2)-C(7)-C(6)	122.2(2)
N(2)-C(7)-H(7)	115.1(16)
C(6)-C(7)-H(7)	122.7(16)
N(1)-C(8)-C(9)	113.56(19)
N(1)-C(8)-H(8A)	108.0(18)
C(9)-C(8)-H(8A)	106.0(18)
N(1)-C(8)-H(8B)	111.6(18)
C(9)-C(8)-H(8B)	109.2(18)
H(8A)-C(8)-H(8B)	108(2)
N(3)-C(9)-C(10)	121.1(2)
N(3)-C(9)-C(8)	115.5(2)
C(10)-C(9)-C(8)	123.2(2)
C(11)-C(10)-C(9)	119.8(2)
C(11)-C(10)-H(10)	121.3(18)
C(9)-C(10)-H(10)	118.9(18)
C(10)-C(11)-C(12)	119.3(2)
C(10)-C(11)-H(11)	124(2)
C(12)-C(11)-H(11)	115(2)
C(13)-C(12)-C(11)	118.4(3)
C(13)-C(12)-H(12)	123.9(16)
C(11)-C(12)-H(12)	117.6(16)
N(3)-C(13)-C(12)	122.8(2)
N(3)-C(13)-H(13)	113(2)
C(12)-C(13)-H(13)	124(2)

Table S4. Anisotropic displacement parameters ($Å^2 \times 10^3$) for $RuCl_2$(dpma)(dmso). The anisotropic displacement factor exponent takes the form: $-2\pi^2[h^2 a^{*2}U^{11} + ... + 2 h k a^* b^* U^{12}]$

	U^{11}	U^{22}	U^{33}	U^{23}	U^{13}	U^{12}
Ru(1)	19(1)	17(1)	14(1)	2(1)	4(1)	5(1)
Cl(1)	20(1)	19(1)	21(1)	4(1)	5(1)	7(1)
Cl(2)	29(1)	23(1)	25(1)	-1(1)	0(1)	0(1)
N(1)	23(1)	25(1)	19(1)	3(1)	5(1)	9(1)
N(2)	19(1)	16(1)	16(1)	1(1)	5(1)	3(1)
N(3)	24(1)	18(1)	19(1)	3(1)	10(1)	5(1)
S(1)	30(1)	28(1)	16(1)	4(1)	9(1)	12(1)
O(1)	50(1)	51(1)	21(1)	16(1)	17(1)	27(1)
C(14)	35(2)	39(2)	27(1)	5(1)	18(1)	8(1)
C(15)	43(2)	34(1)	29(1)	-7(1)	12(1)	10(1)
C(1)	30(2)	41(2)	18(1)	1(1)	-2(1)	10(1)
C(2)	17(1)	32(1)	22(1)	2(1)	4(1)	8(1)
C(3)	18(1)	18(1)	18(1)	2(1)	4(1)	1(1)
C(4)	20(1)	24(1)	23(1)	-2(1)	7(1)	4(1)
C(5)	24(1)	25(1)	22(1)	-5(1)	9(1)	0(1)
C(6)	23(1)	26(1)	17(1)	1(1)	5(1)	1(1)
C(7)	19(1)	22(1)	16(1)	0(1)	4(1)	1(1)
C(8)	31(2)	25(1)	32(1)	11(1)	7(1)	11(1)
C(9)	27(1)	20(1)	25(1)	6(1)	11(1)	6(1)
C(10)	38(2)	20(1)	42(2)	6(1)	21(1)	8(1)
C(11)	41(2)	20(1)	51(2)	-5(1)	22(1)	-6(1)
C(12)	30(2)	25(1)	41(2)	-4(1)	10(1)	-6(1)
C(13)	24(1)	22(1)	28(1)	2(1)	9(1)	2(1)

Table S5. Hydrogen coordinates (× 10^4) and isotropic displacement parameters (Å^2 × 10^3) for $RuCl_2$(dpma)(dmso).

	x	y	z	U(eq)
H(14A)	4590(30)	5150(30)	4517(13)	45(10)
H(14B)	4610(20)	5410(30)	3971(12)	38(8)
H(14C)	4850(20)	3880(40)	4213(11)	42(9)
H(15A)	5710(20)	7130(30)	4902(11)	37(8)
H(15B)	6730(30)	7480(40)	4742(12)	63(11)
H(15C)	5760(20)	7750(30)	4363(10)	33(8)
H(1A)	8987(18)	3560(30)	4712(9)	18(6)
H(1B)	8730(20)	5230(30)	4570(11)	33(8)
H(1C)	8050(20)	4270(30)	4826(10)	28(7)
H(2A)	8994(19)	3030(30)	3795(9)	27(7)
H(2B)	8910(20)	4770(30)	3815(11)	30(8)
H(4)	8920(20)	2830(30)	2870(9)	25(7)
H(5)	8051(18)	3120(30)	2059(9)	22(7)
H(6)	6718(19)	4550(30)	1914(10)	24(7)
H(7)	6170(20)	5450(30)	2603(10)	27(7)
H(8A)	7360(20)	2300(30)	4470(11)	41(9)
H(8B)	7950(20)	1670(30)	4131(10)	27(7)
H(10)	6740(20)	-120(30)	3745(10)	28(8)
H(11)	5320(20)	-430(40)	3154(12)	46(9)
H(12)	4356(19)	1400(30)	2891(10)	24(7)
H(13)	4990(20)	3700(30)	3156(10)	29(7)

Table S6. Torsion angles [°] for $RuCl_2$(dpma)(dmso).

N(1)-C(8)-C(9)-N(3)	-28.0(3)
N(1)-C(2)-C(3)-N(2)	34.3(3)
