# Supplementary figures and images for: Crystal structure of cis,fac-{N,N-bis­[(pyridin-2-yl)meth­yl]methyl­amine-κ3 N,N′,N′′}di­chlorido­(dimethyl sulfoxide-κS)ruthenium(II)
Source: Acta Crystallogr E Crystallogr Commun. 2015 Aug 22;71(Pt 9):m169–70. doi: 10.1107/S2056989015014875 (PMC4555425; doi:10.1107/S2056989015014875)

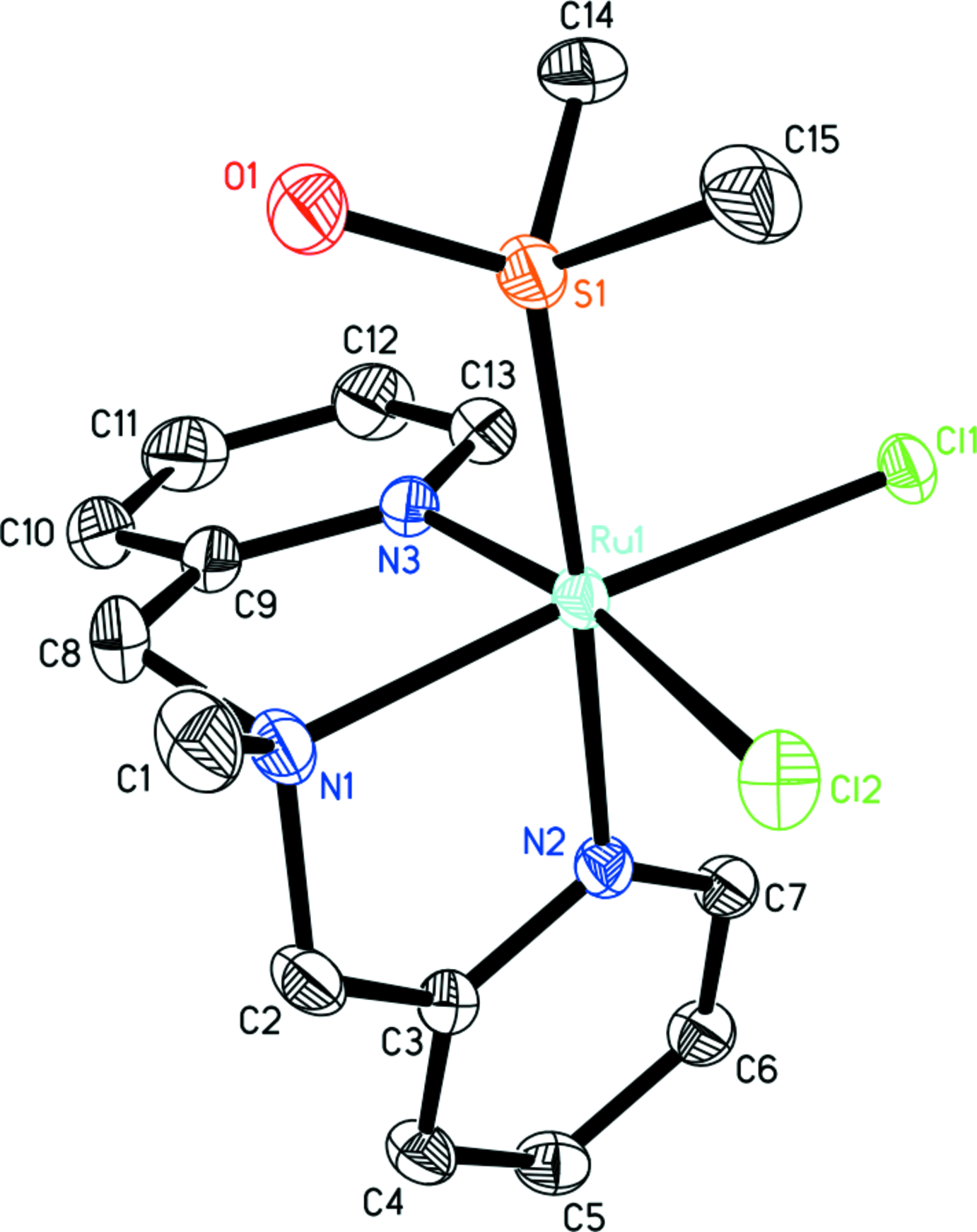

Supplement: Supplementary file 5 [file e-71-0m169-fig1.tif]
